# Effects of Gellan Oligosaccharide and NaCl Stress on Growth, Photosynthetic Pigments, Mineral Composition, Antioxidant Capacity and Antimicrobial Activity in Red Perilla

**DOI:** 10.3390/molecules24213925

**Published:** 2019-10-30

**Authors:** Piotr Salachna, Monika Grzeszczuk, Edward Meller, Małgorzata Mizielińska

**Affiliations:** 1 Department of Horticulture, West Pomeranian University of Technology, 3 Papieża Pawła VI Str., 71-459 Szczecin, Poland; monika.grzeszczuk@zut.edu.pl; 2 Department of Soil Science, Grassland Management and Environmental Chemistry, West Pomeranian University of Technology, 17 Słowackiego Str., 71-434 Szczecin, Poland; edward.meller@zut.edu.pl; 3 Center of Bioimmobilisation and Innovative Packaging Materials, West Pomeranian University of Technology, 35 Janickiego Str., 71-270 Szczecin, Poland; malgorzata.mizielinska@zut.edu.pl

**Keywords:** *Perilla frutescens*, gellan gum, sodium chloride, stress mitigation, biostimulants

## Abstract

The growing market demand for plant raw materials with improved biological value promotes the extensive search for new elicitors and biostimulants. Gellan gum derivatives may enhance plant growth and development, but have never been used under stress conditions. Perilla (*Perilla frutescens*, Lamiaceae) is a source of valuable bioproducts for the pharmaceutical, cosmetic, and food industries. However, there is not much information on the use of biostimulators in perilla cultivation. In this work we investigated the effects of oligo-gellan and salt (100 mM NaCl) on the yield and quality of red perilla (*P. frutescens* var. *crispa* f. *purpurea*) leaves. Plants grown under stress showed inhibited growth, smaller biomass, their leaves contained less nitrogen, phosphorus, potassium, total polyphenol and total anthocyanins, and accumulated considerably more sodium than control plants. Treatment with oligo-gellan under non-saline conditions stimulated plant growth and the fresh weight content of the above-ground parts, enhanced the accumulation of nitrogen, potassium, magnesium and total polyphenols, and increased antioxidant activity as assessed by DPPH and ABTS assays. Oligo-gellan applied under saline conditions clearly alleviated the stress effects by limiting the loss of biomass, macronutrients, and total polyphenols. Additionally, plants pretreated with oligo-gellan and then exposed to 100 mM NaCl accumulated less sodium, produced greater amounts of photosynthetic pigments, and had greater antioxidant activity than NaCl-stressed plants. Irrespective of the experimental treatment, 50% extract effectively inhibited growth of *Escherichia coli* and *Staphylococcus aureus*. Both microorganisms were the least affected by 25% extract obtained from plants untreated with either NaCl or oligo-gellan. In conclusion, oligo-gellan promoted plant growth and enhanced the quality of red perilla leaves and efficiently alleviated the negative effects of salt stress.

## 1. Introduction

The unique physical, chemical, and biological properties of natural polysaccharides and oligosaccharides make them an important source of effective growth promoters and elicitors [1,2,3]. They are also non-toxic, fully biodegradable, and human and environment friendly [4,5,6]. Numerous studies have demonstrated that depolymerized forms of selected polysaccharides may positively affect plant growth and yield [7,8], interfere specifically with many physiological and metabolic processes [9], and induce defense mechanisms under stress [10,11,12].

Gellan gum is an exopolysaccharide produced during aerobic fermentation by *Sphingomonas elodea* bacteria growing in sugar-rich media [13]. Its basic structural unit comprises two glucose residues connected by 1,3 and 1,4 bonds, a single residue of glucuronic acid connected by 1,3 bond, and a single residue of rhamnose connected by different 1,4 bonds [14]. Gellan gum used in in vitro cultures as a medium stimulates development of somatic embryos and plant regeneration [15,16]. Gellan gum and its depolymerized form (oligo-gellan) used for coating of *Eucomis bicolor* L’Her. and *Eucomis comosa* Hort. ex Wehrh. bulbs positively affected plant growth and flowering, improved photosynthetic efficiency with mineral nutrition, and enhanced antimicrobial properties. The effects were more pronounced for oligo-gellan [17].

Salinity is one of the major abiotic stress factors deteriorating crop yield worldwide, and its impact may further increase as the global climate change advances [18]. Salt stress is due to the excessive presence of NaCl or other salts [19]. Substrate salinity inhibits growth and development, disturbs ion management, and disrupts physiological and metabolic processes [20,21]. Plant resistance to salinity involves either the removal of excessive salt from the cells so that the protoplasm is not exposed to its negative effects, or tolerance to toxic and osmotic consequences of increased ion concentration [22,23]. Harmful effects of salinity on plant growth may be reduced by using various growth regulators and biostimulants [24,25,26] that may improve both quantity and quality of crop yield [27,28]. Research studies show that some oligosaccharides used as biostimulants alleviate the negative impacts of stress on plant growth [29,30,31]. It therefore seems interesting to investigate the effects of oligo-gellan in plants exposed to environmental stress.

Perilla (*Perilla frutescens* (L.) Britton), a species native to Southeast Asia and belonging to the Lamiaceae family, provides valuable raw materials for folk medicine, pharmacology, and herbal medicine, as well as for dyeing and the cosmetic industry [32]. Perilla leaves have a pleasant sweet taste and are used as fresh vegetables with several health benefits [33]. In addition to its medicinal properties, perilla is cultivated in many regions in gardens and green areas as an attractive ornamental plant with decorative leaves [34]. *P. frutescens* extracts show anti-inflammatory, anti-allergic and anticancer properties [35,36,37]. Moreover, the herb also exhibits antimicrobial, antipyretic, antiseptic, antispasmodic, antitussive, carminative, expectorant, stomachic, tonic, neuropharmacological, vasodilative and antidepressant-like properties [38,39,40,41]. On the market, perilla cultivars are usually distinguished based on green and red color of their leaves [42,43]. The anthocyanin-rich chemotype (red perilla) is an abundant source of antioxidants and food colorants [44]. The beneficial medicinal effects of perilla organs result from the composition and high amount of volatile and non-volatile biologically active compounds, mainly flavonoids, anthocyanins, phenols, and carotenoids [38,45].

Perilla response to salt stress triggered by NaCl has not been extensively studied. According to Zhang et al. [46], increasing concentrations of NaCl (0–250 mM) clearly inhibit seed germination and seedling growth and increase the accumulation of osmoprotectants in *P. frutescens* varieties. A study by Rouphael et al. [43] demonstrated that although mild (10 mM NaCl) and moderate (20 and 30 mM NaCl) salinity decreased *P. frutescens* var. *frutescens* and *P. frutescens* var. *crispa* yield, it was also perceived as eustress that enhances the content of secondary metabolites. The research literature lacks data on perilla response to higher doses of NaCl and biostimulants during cultivation. 

This is the first paper discussing plant reaction to low-molecular-weight oligo-gellan under specific environmental stress conditions. Our research hypothesis assumed that oligo-gellan would alleviate the effects of NaCl stress in red perilla. We investigated the effects of oligo-gellan and salinity on plant growth; the content of photosynthetic pigments, nutrients, total polyphenols, total anthocyanins, and antioxidants; and the antimicrobial activity of perilla leaf extracts.

## 2. Results

### 2.1. Growth Parameters 

Data presented in Table 1 show significant effects of oligo-gellan on plant growth and biomass accumulation. Compared with the control group, oligo-gellan increased plant height, fresh weight of above-ground part, leaves, and shoots by 10.3%, 7.5%, 10.9%, and 9.3%, respectively. Plant height, number of shoots, fresh weight of above-ground part, leaves, and shoots were significantly inhibited in the presence of 100 mM NaCl and dropped by 11.2%, 9.9%, 21.7%, 28.3%, and 20.1%, respectively, in comparison with the control group. Oligo-gellan clearly alleviated NaCl stress by improving plant height, number of shoots, fresh weight of above-ground part, leaves, and shoots by 13.1%, 18.6%, 16.6%, 30.3%, and 40.0%, respectively, when compared with salt stress variant without oligo-gellan treatment (Table 1).

### 2.2. Photosynthetic Pigments 

**Figure 1:**
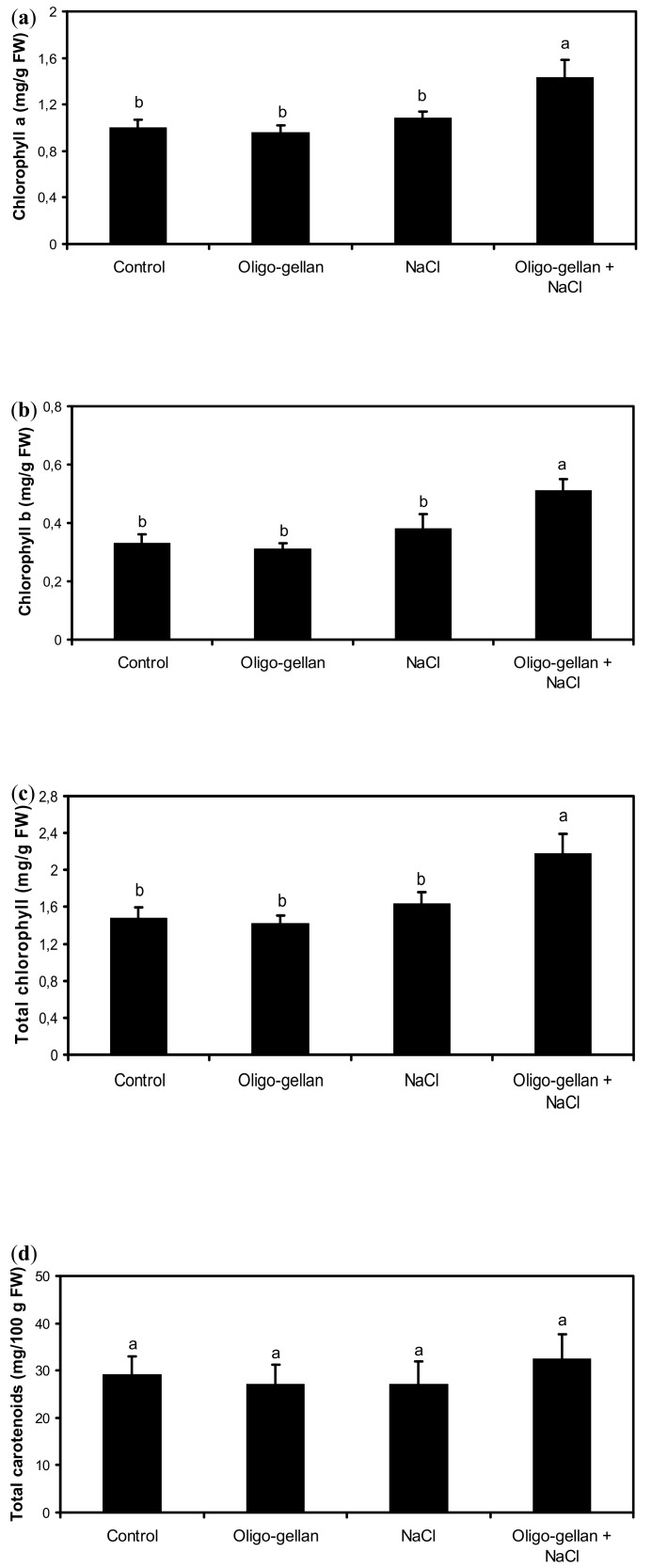
Effects of oligo-gellan pretreatment (100 ppm) and salt stress (100 mM NaCl) on concentrations of chlorophyll a (**a**); chlorophyll b (**b**); total chlorophyll (**c**); and total carotenoids (**d**) in red perilla plants. Bars represent the means of three biological replicates ± standard deviations. Different letters indicate statistical differences at *p* ≤ 0.05 (Tukey’s test).

Plant exposure to either oligo-gellan or NaCl did not alter chlorophyll content, but their joint application brought about significant changes in this parameter (Figure 1a–c). In NaCl-stressed plants treated with oligo-gellan, the levels of chlorophyll a, chlorophyll b, and total chlorophyll increased by 43.0%, 54.5%, and 47.3%, respectively, as compared with untreated plants. Leaf carotenoid content was independent of either oligo-gellan or NaCl treatment (Figure 1d).

### 2.3. Mineral Composition

The nutritional status of red perilla changed in the presence of oligo-gellan and NaCl (Table 2). In non-stress conditions, oligo-gellan enhanced the leaf content of nitrogen (17.3%), potassium (15.6%), and magnesium (12.0%), while the levels of phosphorus and sodium were not affected. Salt stress visibly reduced the content of nitrogen (24.1%), phosphorus (21.6%), and potassium (18.6%), and increased the level of sodium by 8.5 times. Plants exposed to NaCl and treated with oligo-gellan accumulated less sodium and more nitrogen, phosphorus, potassium, and magnesium than those treated with NaCl but not with oligo-gellan. The K/Na ratio dropped considerably in the plants experiencing stress, regardless the oligomer treatment (Table 2).

### 2.4. Total Polyphenols, Total Anthocyanins, and Antioxidant

**Figure 2:**
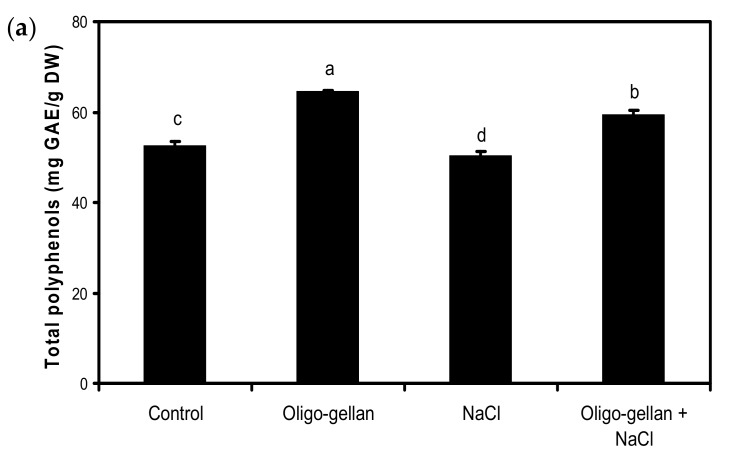

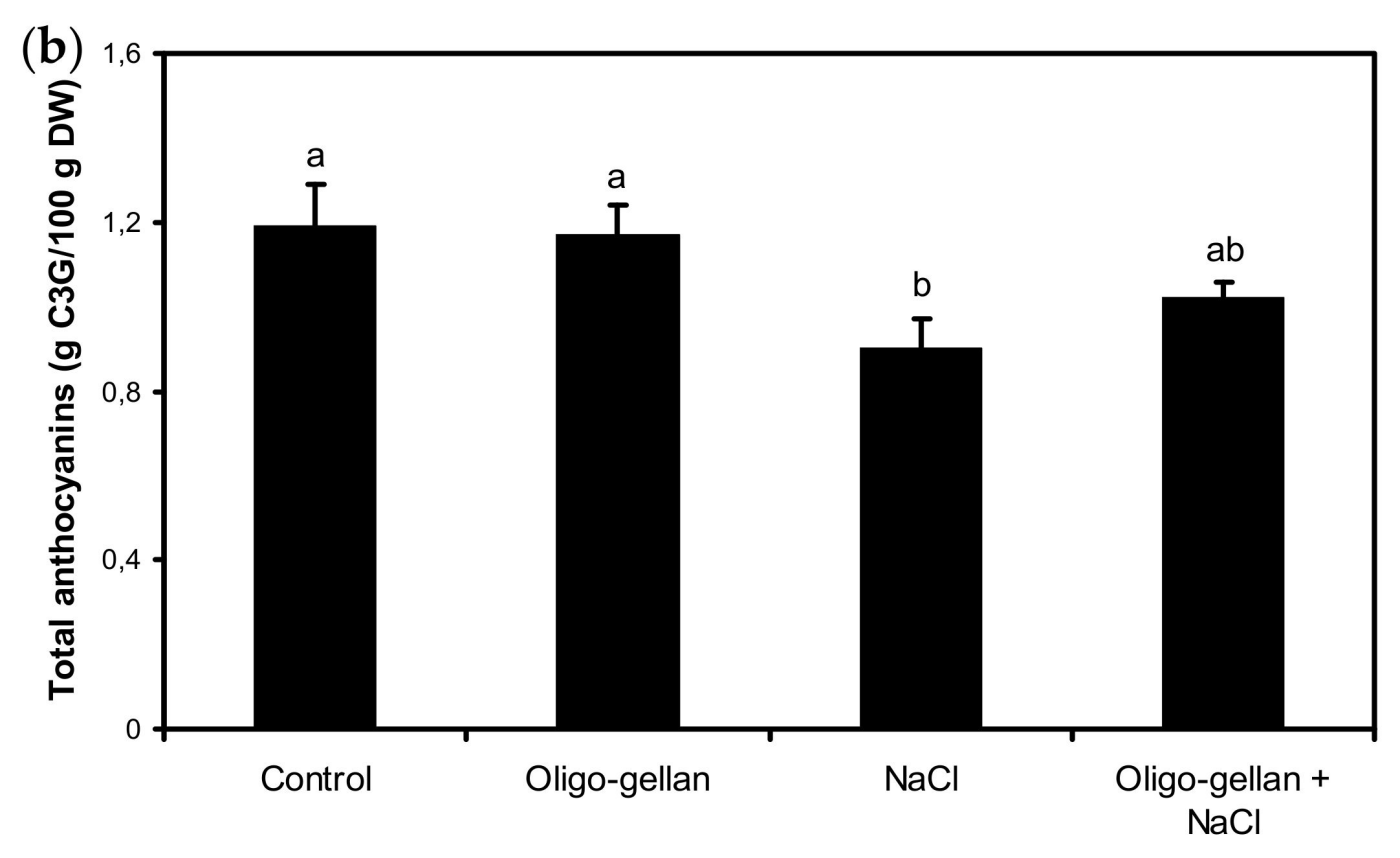
Effects of oligo-gellan pretreatment (100 ppm) and salt stress (100 mM NaCl) on total polyphenols (**a**) and total anthocyanins (**b**) of red perilla plants. Bars represent means of three biological replicates ± standard deviations. Different letters indicate statistical differences at p ≤ 0.05 (Tukey’s test).

**Figure 3:**
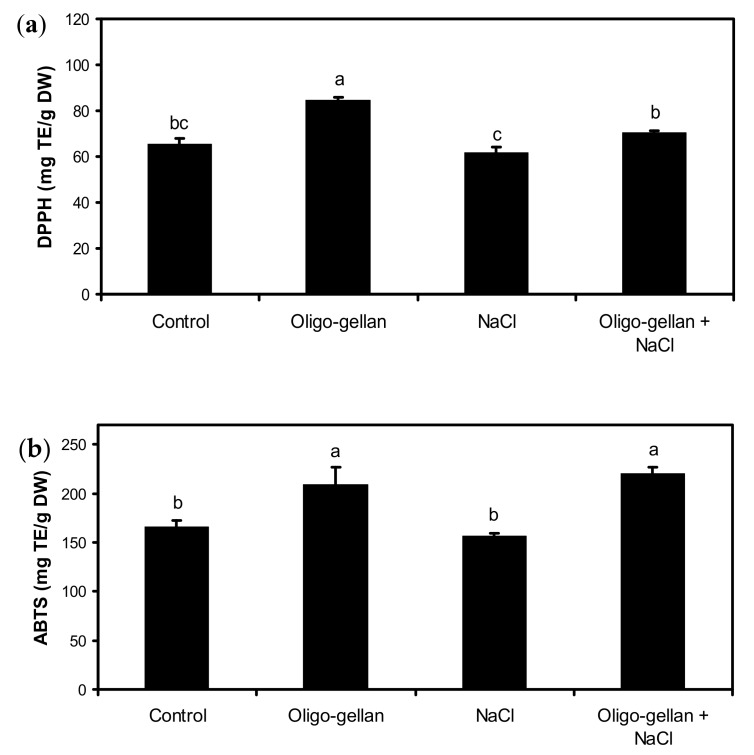
Effects of oligo-gellan pretreatment (100 ppm) and salt stress (100 mM NaCl) on antioxidant activity of red perilla plants assessed by 2,2-diphenyl-1-picrylhydrazyl (DPPH) (**a**) and 2,2′-azino-bis(3-ethylbenzothiazoline-6-sulfonic acid (ABTS) (**b**). Bars represent means of three biological replicates ± standard deviations. Different letters indicate statistical differences at *p* ≤ 0.05 (Tukey’s test).

Data presented in Figure 2a and Figure 3a,b indicate that oligo-gellan treatment increased the leaf content of total polyphenols by 23.1%, antioxidant activity assessed by DPPH by 28.9%, and ABTS by 25.4% as compared with control. In plants exposed to NaCl, the content of total anthocyanins dropped by 24.4% vs. control (Figure 2b). Salt also reduced the levels of total polyphenols and antioxidant activity measured by DPPH. Oligo-gellan treatment limited the negative effects of salt on the accumulation of secondary metabolites. The plants treated with both oligo-gellan and NaCl featured a greater content of total polyphenols (18.3%), total anthocyanins (13.3%), DPPH (13.9%), and ABTS (41.1%) than NaCl-stressed plants.

### 2.5. Antimicrobial Activity

This study clearly demonstrated that perilla acetone extracts exerted considerable influence on the viability of *Staphylococcus aureus* strain. The activity of the extract depended on its concentration. Bacterial growth was completely inhibited by 50% extract, while a medium containing 25% acetone extract from non-treated plant caused an average 3 log decrease in the number of microbial cells. Plant treatment with oligo-gellan or/and NaCl also affected the antimicrobial activity of the plant extracts. *S. aureus* growth ceased completely on the media containing 25% perilla extract from oligo-gellan- or NaCl-treated plants. This confirms that plant exposure to oligo-gellan or NaCl increased the antibacterial activity of extracts against this strain. Plant treatment with oligo-gellan and NaCl also increased the antimicrobial activity of the extracts in comparison with non-treated control, but decreased the extract activity in comparison with separate treatments with either oligo-gellan or NaCl (Table 3). 

Results also indicate that perilla acetone extracts also strongly influenced the viability of *Escherichia coli* strain in a concentration-dependent manner. Similarly, as for *S. aureus*, 50% extract caused a complete inhibition of bacterial growth, while the medium containing 25% extract from non-treated plants caused an average 4 log decrease in the number of *E. coli* cells. Plant treatment with either oligo-gellan or NaCl had no significant influence on the antimicrobial activity of the extracts, but combined application of oligo-gellan and NaCl enhanced their antimicrobial properties (Table 3).

## 3. Discussion

Biostimulants enhance natural plant resistance/tolerance to abiotic stress, and in non-stress conditions they facilitate the fulfillment of plant genetic potential [47,48,49]. The number of new biostimulants reported in the literature is constantly growing [50,51], and biodegradable and bioactive degraded polysaccharides stand out as a highly promising group [52,53]. Our study focused on derivatives of exopolysaccharide gellan gum and their biostimulating properties. The experimental object was red perilla drenched with a solution of low-molecular-weight oligo-gellan and then a 100-mM solution of NaCl. Salinity considerably inhibited plant growth, reduced the content of macronutrients, caused excessive accumulation of sodium, and resulted in unfavorable K/Na ratio. Toxic excess of sodium and lowered K/Na ratio may disturb the absorption of other ions and their transport to above-ground parts, and consequently negatively affect plant growth and metabolism [22,24]. Our findings partially corroborate those published by Rouphael et al. [43], where moderate salinity lowered plant biomass and nitrate content and enhanced sodium levels in *P. frutescens* var. *crispa*. The authors also found that application of 10 and 20 mM NaCl increased the content of total polyphenols, the level of which remained unaffected by 30 mM NaCl. In our study, 100 mM NaCl lowered the levels of total polyphenols and total anthocyanins, which might be due to physiological and biochemical changes resulting from previously discussed unfavorable K/Na ratio. It may be assumed that low doses of NaCl stimulate and high doses inhibit polyphenol biosynthesis in perilla plants. Similar relationships were reported by Zhou et al. [54] in *Schizonepeta tenuifolia* Briq. (Lamiaceae), who detected the highest levels of total phenolics and flavonoids in plants treated with 25 mM NaCl, and the lowest levels in plants exposed to 100 mM NaCl. Changes in the levels of polyphenols triggered by salinity may be due to oxidative stress involving and increased production of reactive oxygen species (ROS) that may severely disturb plant metabolism [55,56]. 

In this study, oligo-gellan demonstrated a multidirectional biostimulatory activity towards red perilla. Plants exposed to its presence had greater weight of the above-ground part and accumulated more nutrients. Enhanced biomass may result from the intensified uptake of macronutrients necessary for proper plant development, as also reported in *Eucomis bicolor* and *E. comosa* treated with oligo-gellan [17]. The biostimulant applied in red perilla increased total contents of polyphenols and antioxidant activity as assessed by DPPH and ABTS assays. Intensified antiradical activity probably reflected the enhanced content of polyphenols. We noticed similar relationships in *Ornithogalum saundersiae* Bak., where coating bulbs in gellan gum and chitosan complexes increased the leaf content of total phenolics, ascorbic acid, and antioxidant activity [57]. Greater accumulation of total phenolics and other secondary metabolites in response to carrageenan and chitosan oligomers was also reported in *Mentha × piperita* L. [6,9]. Shukla et al. [58] and González et al. [59] claimed that oligomeric molecules of polysaccharide derivatives might bind to different membrane receptors and initiate gene expression, leading to increased hormone and enzyme biosynthesis which in turn increases cell division and secondary metabolite production in plants. 

Our results show that oligo-gellan alleviated the effects of substrate salinity. In the presence of excessive salt (100 mM NaCl), red perilla plants treated with oligo-gellan showed lower sodium accumulation and smaller biomass decline than plants exposed to salt and not treated with the biostimulant. At the same time, application of oligo-gellan increased the accumulation of macronutrients, chlorophylls, and polyphenols, and intensified antioxidant activity. It may be concluded that greater availability of macronutrients and chlorophylls positively affected photosynthesis intensity and consequently plant growth. These outcomes indicate that oligo-gellan treatment under salt stress triggers biochemical and physiological changes in the leaves that may be responsible for improved plant tolerance to stress through effective antioxidant defense. These claims are substantiated by the work of Zou et al. [60], who reported that chitooligosaccharides reduced the negative effects of salinity in *Triticum aestivum* L. seedlings by increasing the content of chlorophyll and soluble sugars and enhancing the activity of antioxidant enzymes (CAT, POD, and SOD).

The study clearly demonstrated that acetone extracts of red perilla possess promising antibacterial properties against *S. aureus* and *E. coli* strains. Similar results were reported by Kang et al. [61], who noted that *Perilla frutescens* extracts inhibited a broad range of Gram-negative and Gram-positive bacteria. As mentioned by Yamamoto and Ogawa [62], ethyl acetate and ethanolic extracts of *P. frutescens* showed remarkable activity against *Porphyromonas gingivalis*, *Streptococcus mutans*, *S. sobrinus*, *S. salivarius*, *S. oralis*, *S. mitior*, and *S. sanguis*. Moreover, crude aqueous extracts of *P. frutescens* exhibited significant antibacterial action against *E. coli* O157:H7 [63]. Our results also support the general claim that the concentrations of the extract, oligo-gellan, and/or NaCl determine the antibacterial activity of red perilla extracts. In our previous study [17], the influence of gellan gum and oligo-gellan treatment on the antimicrobial activity of *E. comosa* and *E. bicolor* bulb extracts was evaluated. Acetone extracts from the bulbs treated with gellan gum and oligo-gellan more effectively reduced the count of *Bacillus atrophaeus*, *E. coli*, and *S. aureus* than those from the bulbs not treated with the polysaccharide.

## 4. Materials and Methods 

### 4.1. Characterization of Depolymerized Gellan Gum

Oligo-gellan was prepared by the acid hydrolysis of gellan gum. The molecular mass of oligo-gellan (56,000 g mol^−1^) was determined by HPSEC using an S1000 pump, an S2300 refractive index detector, and a 20 µL sample loop (Knauer, Berlin, Germany). The oligomer was described in detail in our earlier work [17].

### 4.2. Plant Material and Experimental Conditions

Perilla *(Perilla frutescens* var. *crispa* f. *purpurea*) seeds (W. Legutko Breeding and Seed Company, Jutrosin, Poland) were used in our study. The plants were grown under natural photoperiod from March until August in a greenhouse belonging to the Department of Horticulture, West Pomeranian University of Technology in Szczecin (53° 25′ N, 14° 32′ E). Air temperature was controlled with vents that opened automatically when it exceeded 22 °C during the day and 18 °C at night. The seeds were broadcasted into plastic boxes, and then five-week-old seedlings of similar size were transplanted individually into 21-cm-diameter round plastic pots with a volume of 5 dm^3^. A substrate consisting of deacidified peat (Kronen, Poland), pH 6.0, was supplemented with a multicomponent Hydrocomplex fertilizer (Yara International ASA, Norway) at a dose of 3 g·dm^−3^, containing 12% N (5% N-NO_3_ and 7% N-NH_4_), 11% P_2_O_5_, 18% K_2_O, 2.7% MgO, 8% S, 0.015% B, 0.2% Fe, 0.02% Mn, and 0.02% Zn.

### 4.3. Treatments 

The experiment was designed as a univariate study in a random block arrangement. The plants were divided into four groups:

1. Non-treated controls;
2. Plants drenched with oligo-gellan solution;
3. Plants drenched with NaCl solution;
4. Plants drenched first with oligo-gellan and then with NaCl.

Drenching with oligo-gellan at 100 ppm started 14 days after transplantation (DAT). The plants were drenched a total of eight times, every five days, and each time 100 cm^3^ of the solution was applied. Starting at 55 DAT the plants were exposed to salt stress consisting of drenching with 100 mM NaCl solution. Four salt treatments were performed, one every five days, with 200 cm^3^ of NaCl solution per Plant Oligo-gellan and NaCl concentrations were determined based on a pilot experiment. Control plants were watered exclusively with tap water of electrolytic conductivity 0.45 mS cm^−1^. Each treatment included three repetitions, and each repetition consisted of eight plants plus border plants.

### 4.4. Determination of Growth Parameters

Plant height, number of shoots, fresh weight of above-ground part, shoots, and leaves per plant were determined in plants of similar size on the last day of culture, i.e., at 137 DAT. The plants were then entering the blooming stage. The leaves collected from upper external parts of the plants (20–25 leaves per plant) were dried up in darkness for three weeks at 25–30 °C.

### 4.5. Estimation of Chlorophyll and Carotenoid Content

In fresh leaves collected on the last day of cultivation, the content of chlorophylls and carotenoids was evaluated as described by Lichtenthaler and Wellburn [64] with some modifications. Approximately 0.5 g of a homogenized sample was weighed and blended with a few drops of 80% acetone and then mixed with 80% acetone in a measuring flask of 50 mL volume. The flask was placed in an ultrasonic cleaner for 5 min. Then, the mixture was transferred to a tube and centrifuged at 10,000 rpm for 10 min, at 10 °C. Absorbance of the samples was determined at 441, 646, 652, and 663 nm against a blank sample (80% acetone). The concentrations of chlorophyll a, chlorophyll b, total chlorophyll and total carotenoids were calculated based on the following equations:

chlorophyll a (mg/g FW) = (12.21 × *E*663 – 2.81 × *E*646) × (V /1000 × m), (1)

chlorophyll b (mg/g FW) = (20.13 × *E*646 – 5.03 × *E*663) × (V /1000 × m), (2)

total chlorophyll (mg/g FW) = (27.8 × *E*652) × (V /1000 × m), (3)

total carotenoids (mg/100g FW) = [ (1000 × *E*_441_) − 3.27 × (12.21 × *E*_663_ − 2.81 × *E*_646_) − 104 × (20.13 × *E*_646_ − 5.03 × *E*_663_) ] × [V/1000 × (m × 229)], (4)

where *E* is the absorbance at a specific wavelength, V is the volume of a measuring flask in mL, and m is the weight of a sample in g.

### 4.6. Macronutrient Analysis

The leaves dried up to dry weight and ground were mineralized in 96% H_2_SO_4._ Total nitrogen content was determined using Kjeldahl method, phosphorus content by colorimetric method according to Barton, and potassium and sodium by atomic absorption spectrometry (ASA). The analyses were performed as described by Ostrowska [65], and mineral content was expressed as percent of dry weight.

### 4.7. Determination of Total Anthocyanin Content

#### 4.7.1. Anthocyanin Extraction

The extraction procedure followed the method described by Anuar et al. [66]. One gram of homogenized raw plant material was mixed with 50 mL of methanol acidified with 0.5% acetic acid. After extraction in a thermostatic water bath (60 min, 60 °C) the mixtures were centrifuged at 1500 rpm for 10 min and then filtered (No. 1 Whatman paper). Extractions were done in triplicate.

#### 4.7.2. Anthocyanin Content Determination

Total anthocyanin content was determined according to the pH differential spectrophotometric method [67]. Ten milliliters of extracts were diluted in 50 mL of two different buffers: 0.025 M potassium chloride pH 1.0 and 0.4 M sodium acetate pH 4.5. After 15 min of incubation at room temperature, absorption was measured at 520 and 700 nm using a spectrophotometer and calculated as *A* = [(*A*_520_ − *A*_700_) pH 1.0 − (*A*_520_ − *A*_700_) pH 4.5]. Total anthocyanin content was expressed as cyanidin-3-glucoside (C3G) equivalents per liter of concentrate according to the following equation:

[*A* × MW × DF × V × 100] / [*ε* × *λ* × m] (5)

where: *A* is absorbance, MW is the molecular weight of cyanidin-3-glucoside (449.2 g/mol), DF is the dilution factor, V is the solvent volume (mL) that was brought as sample stock solution, *ε* is the molar absorptivity (26,900), *λ* is the cuvette path length (1 cm), and m is the sample weight (g). Total anthocyanin content was expressed as g C3G/100 g DW.

### 4.8. Estimation of Total Polyphenol Content and Antioxidant Activity

#### 4.8.1. Preparation of Plant Extracts

Preparation of plant extracts followed the method proposed by Wojdyło et al. [68] with some modifications. A sample of 1 g dried (through-flow laboratory dryer, 35 °C) ground leaves was supplemented with 80% aqueous methanol (MeOH) up to 100 mL volume. The mixtures were placed in an ultrasonic bath at room temperature and sonicated for 30 min (2 × 15 min) and then left for 24 h at room temperature (~20 °C). The obtained extracts were filtered over Whatman No. 1 filter paper. The filtrates were centrifuged at 1500 rpm for 10 min. All the extractions were carried out in triplicate. The extracts were kept at 4 °C and used for the analyses within 24 h.

#### 4.8.2. Total Polyphenol Content

Total polyphenol content was analyzed spectrophotometrically using the Folin–Ciocalteu colorimetric method as described by Wojdyło et al. [68]. Plant extract (100 µL) was mixed with 0.2 mL of the Folin–Ciocalteu reagent, 2 mL of deionized water and 1 mL of 20% sodium carbonate. The samples were allowed to stand for 1 h at room temperature in darkness. The absorbance was read at 760 nm. Gallic acid (GAE) was used to calculate the standard curve, and the results were expressed as GAE milligrams per g of DW.

#### 4.8.3. Determination of DPPH Radical Scavenging Capacity

Antioxidant activity of *P. frutescens* leaves towards DPPH (2,2-diphenyl-1-picrylhydrazyl) radical was determined according to the procedures of Wojdyło et al. [68] and Kumaran and Karunakaran [69]. A 1 mL volume of the methanol extract was diluted two times. DPPH (0.3 mM) was dissolved in pure ethanol (99.8%). Plant extract (0.6 mL) was added to 1.8 mL of pure ethanol (EtOH) and 0.6 mL of DPPH solution. The samples were incubated at room temperature for 10 min in the dark. Reduction of DPPH radical was determined spectrophotometrically by measuring the absorbance at 517 nm. For calibration of the standard curve, Trolox (TE, 6-hydroxy-2,5,7,8-tetramethylchromane-2-carboxylic acid) was used, and the results were expressed as mg of TE equivalent per g of the sample DW.

#### 4.8.4. Determination of Free-Radical Scavenging Ability Using a Stable ABTS Radical Cation

The free-radical scavenging activity was determined by ABTS radical cation decolorization procedures described by Wojdyło et al. [68], Re et al. [70], and Chew et al. [71] with some modifications. ABTS (2,2′-azino-bis(3-ethylbenzothiazoline-6-sulfonic acid) diammonium salt was dissolved in deionized water to a 7 mM concentration. ABTS radical cation (ABTS^•+^) was produced by reacting ABTS stock solution with 2.45 mM potassium peroxodisulfate and kept in the dark at room temperature for 16 h before use. The ABTS^•+^ solution was diluted with PBS (phosphate buffered saline, pH 7.4) until its absorbance was equilibrated to 0.7 (±0.02) at 734 nm before usage. Then, 3 mL of diluted ABTS^•+^ solution (A_734_ = 0.7 ± 0.02) was added to 300 µL of methanolic plant extract. The absorbance was read after six minutes. Trolox was used for calibration of the standard curve, and the results were expressed as mg TE per g of the sample DW.

### 4.9. Antibacterial Activity

The microorganisms tested in this study were obtained from the Leibniz Institute, German Collection of Microorganisms and Cell Cultures (DSMZ, Braunschweig, Germany). The investigated organisms included *Staphylococcus aureus* DSMZ 346 and *Escherichia coli* DSMZ 498. Acetone was used to extract the active substance from dry leaves. To verify antimicrobial properties of the extracts, Tryptic Soy Broth (TSB), Tryptic Soy Agar (TSA), Sand MacConkey agar (Merck, Darmstadt, Germany) were used. All media were prepared according to a protocol provided by Merck.

#### 4.9.1. Extract Preparation

Samples of dry leaf (10 g) were extracted with 100 g of 70% aqueous acetone. They were then separately kept in a sonication water bath for one hour. The temperature of the bath was maintained at 15 °C by adding ice. Sonication parameters were: cycle = 0.5; amplitude = 50%. The acetone extracts were concentrated at 40 °C. After evaporation of the aqueous acetone, the extracts were dissolved in 100 g of dimethyl sulfoxide. At the next step of the experiment, the samples were filtered through a 0.2 µm filter (Sartorius AG, Göttingen, Germany). The extracts were used in further analyses.

#### 4.9.2. Antibacterial Analysis

To verify antimicrobial properties of the samples, TSB MacConkey agar and TSA media were prepared. First, the bacterial cells of *S. aureus* and *E. coli* were pre-grown on TSA or MacConkey agar for 24 h at 30 °C. After incubation, the biomass (each strain separately) was suspended in a sterile 0.85% NaCl solution to obtain 1.5 × 10^8^ CFU/mL. After that, TSB medium was prepared and used to assemble 25% and 50% extract solutions (each in 10 mL of TSB). The suspended biomass was added to sterile flasks that contained TSB with extracts at a ratio of 1:10, and further mixed with a magnetic stirrer (250 rpm, Ika) for 15 min. The medium with extract-free biomass served as a control. After stirring, 100 µL of each sample were introduced into the media and incubated at 30 °C for 24 h. Cell concentration was expressed as colony-forming units (CFU) per mL and determined by making serial decimal dilutions and plating on TSA and MacConkey agar.

### 4.10. Statistical Analysis

Statistical analysis of the results was based on one-way analysis of variance (ANOVA). Mean values for the experimental variants were compared with Tukey’s test at significance level *p* ≤ 0.05. All calculations were made with Statistica 13.3 package (TIBCO Software, Palo Alto, CA, USA). Data presented in tables and figures represent means ± standard deviations from independent repetitions (SD; *n* = 3–9).

## 5. Conclusions

Oligo-gellan may be recommended as an effective biostimulant improving growth, yield, and biological quality of red perilla grown under both optimal and salt-stress conditions. This is the first study demonstrating enhanced plant tolerance to salinity conferred by oligo-gellan treatment. Oligo-gellan alleviates the negative effects of salinity by improving plant mineral status, antioxidant content, and antioxidant activity. The study also provided new information on the chemical composition and biological activity of red perilla leaves used as a source of valuable antioxidant and antibacterial compounds with potential health benefits.

## Figures and Tables

**Table 1:** Effects of oligo-gellan pretreatment (100 ppm) and salt stress (100 mM NaCl) on red perilla growth parameters. Mean values in the columns marked with the same letters show no significant difference at *p* ≤ 0.05 (Tukey’s test, *n* = 9).

Treatment	Plant Height (cm)	Number of Shoots per Plant	Fresh Weight of Above-Ground Part (g)	Fresh Weight of Leaves per Plant (g)	Fresh Weight of Shoots per Plant (g)
Control	84.8 ± 4.54 b	37.5 ± 1.75 a	305.8 ± 4.60 b	176.4 ± 3.45 b	105.5 ± 3.72 b
Oligo-gellan	93.5 ± 1.25 a	40.1 ± 1.83 a	328.0 ± 6.68 a	192.8 ± 9.47 a	117.1 ± 4.65 a
NaCl	75.3 ± 0.65 c	33.8 ± 0.99 b	239.6 ± 6.46 d	141.0 ± 4.11 c	75.6 ± 3.97 c
Oligo-gellan + NaCl	85.2 ± 2.00 b	39.4 ± 0.95 a	284.2 ± 8.12 c	191.7 ± 2.59 a	98.5 ± 4.86 b

**Table 2:** Effects of oligo-gellan pretreatment (100 ppm) and salt stress (100 mM NaCl) on macronutrient concentrations and K/Na ratio of red perilla plants. Mean values in the columns marked with the same letters show no significant differences at *p* ≤ 0.05 (Tukey’s test, *n* = 3).

Treatment	N (% DW)	P (% DW)	K (% DW)	Mg (% DW)	Na (% DW)	K/Na
Control	2.20 ± 0.13 b	0.37 ± 0.02 a	2.63 ± 0.12 b	0.25 ± 0.02 b	0.17 ± 0.02 c	15.7 ± 2.53 a
Oligo-gellan	2.58 ± 0.04 a	0.37 ± 0.02 a	3.04 ± 0.13 a	0.28 ± 0.01 a	0.23 ± 0.03 c	13.3 ± 0.74 a
NaCl	1.67 ± 0.12 c	0.29 ± 0.01 b	2.14 ± 0.13 c	0.22 ± 0.01 b	1.45 ± 0.11 a	1.48 ± 0.17 b
Oligo-gellan + NaCl	1.94 ± 0.08 b	0.35 ± 0.01 a	2.84 ± 0.07 b	0.30 ± 0.02 a	1.03 ± 0.08 b	2.78 ± 0.25 b

**Table 3:** Effects of oligo-gellan pretreatment (100 ppm) and salt stress (100 mM NaCl) on the activity of red perilla extracts against *Staphylococcus aureus* and *Escherichia coli*. The number of bacterial cells are presented as an average of three samples with standard deviation.

Treatment	Concentration of Extract (%)	Concentration of Bacterial Cells	
		*S. aureus* (10^3^ CFU/mL)	*E. coli* (10^3^ CFU/mL)
Control	50 25	0 166.00 ± 23.30	0 42.50 ± 17.70
Oligo-gellan	50 25	0 0	0 27.50 ± 4.95
NaCl	50 25	0 0	0 12.00 ± 2.83
Oligo-gellan + NaCl	50 25	0 5.20 ± 1.70	0 9.00 ± 5.66

The number of bacterial cells for control sample: *S. aureus* = 128.00 ± 4.24 × 10^6^ colony-forming units (CFU) per mL; *E. coli* = 6.0 ± 1.7 × 10^8^ CFU/mL.
